# Changes of the Microbiota Composition on the Surface of Pig Carcasses during Chilling and Its Associations with Alterations in Chiller’s Temperature and Air Humidity

**DOI:** 10.3390/foods10092195

**Published:** 2021-09-16

**Authors:** Xiaonan Jia, Yingqun Nian, Di Zhao, Juqing Wu, Chunbao Li

**Affiliations:** Key Laboratory of Meat Processing and Quality Control, Ministry of Education, Key Laboratory of Meat Processing, Ministry of Agriculture and Rural Sciences, Jiangsu Collaborative Innovation Center for Quality Control of Meat Production and Processing, College of Food Science and Technology, Nanjing Agricultural University, Nanjing 210095, China; 2018808097@njau.edu.cn (X.J.); nianyingqun@njau.edu.cn (Y.N.); zhaodi@njau.edu.cn (D.Z.); wujuqing@njau.edu.cn (J.W.)

**Keywords:** pig carcass, chilling, microbiota, drug-resistant genes, air temperature

## Abstract

In this study, we investigated changes of microbiota composition on the surface of pig carcasses during chilling and their associations with temporal and spatial changes of wind speed, air temperature, and air humidity. The composition of microbiota on a carcass surface varied greatly with sampling sites; in particular, the surfaces of forelegs and neck had higher load of microorganisms and different microbiota composition compared to in the air and other carcass parts. However, such a difference in the microbiota composition decreased as chilling time extended. The positive detection ratios of microbial genes resistant to sulfonamides, quinolones, tetracyclines, and β-lactams were found different greatly with chilling time and sampling sites. The β-lactam and tetracycline resistant genes were observed in higher ratios in airborne microorganisms in the chiller, while the sulfa and tetracycline resistant genes had higher ratios in the microbiota on pig carcasses. Actual measurements and dynamic simulation showed that air temperature and humidity varied greatly among different places in a chiller within the first 8 h of chilling, with higher values close to the door, but the differences became smaller afterwards. The micro-environmental differences and changes in the chiller may cause the different composition of microbiota on pig carcasses.

## 1. Introduction

Carcass chilling is a critical step for processing of chilled pork. Low temperature can reduce the growth of spoilage microorganisms on pig carcasses [1]. A chiller is the main place for carcass chilling. The main air supply in a chiller is the forced convection heat exchange, forcing the outside air into the box and performing heat exchange with an evaporator, and as a result, the air temperature is reduced to achieve the purpose of refrigeration [2]. The chilling method plays a critical role in the airflow organization in a chiller [3]. Bad air flow organization may lead to insufficient heat exchange between cold and hot air, resulting in uneven distribution of air temperature and humidity [4]. Air temperature is one of the most important factors to inhibit the growth of microorganisms, and uneven temperature may help to increase the growth of microorganisms in a chiller. In addition, the difference in wind speed in a chiller affects the contact area between the cold wind and pig carcasses and the growth of microorganisms. Therefore, the size, temperature, humidity, and wind speed of a chiller affect the carcass chilling efficiency and meat contamination [5,6]. At a high wind speed, the surrounding environment of a carcass has high humidity and low temperature. When a chiller is full of pig carcasses, the wind speed and air temperature decline rapidly, but the air humidity increases, and finally, the wind speed, air temperature, and humidity tend to be stable [7].

Microbial contamination is a main factor for causing meat deterioration [8], which is characteristic of unacceptable discoloration, unpleasant odor, and slime formation [9]. The microbial spoilage of meat is a complex process that is affected by competition between different microbial populations and their biological and abiotic interactions [10] under processing and storage conditions [11]. Therefore, detecting the microbial population and structure in meat plays an important role in maintaining meat quality and extending shelf life.

The inappropriate use of antibiotics to promote animal growth may cause drug resistance in microorganisms. Animals and farming environments are reservoirs of drug-resistant bacteria [12]. Sulfa/trimethoprim, tetracyclines, macrolides, penicillins, and aminoglycosides are the most widely used antibiotics [13,14]. The drug-resistant genes are usually detected in farm animals [15,16,17,18]. Tetracyclines and sulfa resistant genes are the most common resistance genes in animal feces and urine [15,19], and microorganisms acquire antibiotic resistance by carrying related genes [15]. These microorganisms may spread from the environment to humans [20,21]. However, few data are available on the resistant genes of microorganisms existing on the surface of pig carcasses.

The objectives of this study were to investigate the population structure and drug-resistant genes of microorganisms on the surface of pig carcasses and the changes in wind speed, temperature, and humidity in a chiller during chilling of pig carcasses.

## 2. Materials and Methods

### 2.1. Chiller Selection

The experiment was performed in a local pig slaughterhouse from June to August 2019. The size of a chiller is 11.4 m × 7.7 m × 4.8 m, which has eight tracks. Each track can hang a maximum of 40 pig carcasses. Two air coolers are installed above the track (approximately 4 m above the ground).

### 2.2. Simulation of Wind Speed, Temperature and Humidity in the Chiller

The chiller was full of three hundred pig carcasses within 8 h. The wind speed, temperature, and humidity of the chiller were measured at 0, 2, 4, 6, 8, 10, 12, and 14 h. Then the Ansys 16.0 system (ANSYS, Canonsburg, PA, USA) was used to divide into a number of grids to calculate the wind speed, temperature, and humidity, and the simulation graphs were obtained.

The forced closed air circulation in a cooler belongs to the large space turbulent heat exchange, and the following assumptions are made: (1) The air in the chiller conforms to Boussineq’s assumption and is not compressible; (2) The air flow in the chiller is a steady-state turbulent flow [22].

Since the forced convection circulation flow field driven by a fan in the chiller is a high Re number turbulent flow, it is solved by the standard k-ε equation. However, due to the low Re number in the viscous bottom layer close to the wall, the wall function must be corrected [23].

The actual velocity of the air outlet of the fan was measured by a thermal anemometer. The chiller door was the pressure inlet, and the pressure was set to 0. The temperature and humidity of the air outlet of the air cooler were measured by a temperature and humidity meter (Biaozhi GM1362, Jumaoyuan, Shenzhen, China). The fluid density was taken from the air density (ρ = 1.225 kg/m^3^) [22], and the average flow velocity (15–20 m/s), and the hydrodynamic viscosity were based on the aerodynamic viscosity (μ = 0.000017 kg/m·s) [22]. The orbital thermal conductivity was set at 16.27 w/(m·k), and the thermal conductivity of pig carcasses was set at 2 w/(m·k). Within the first 8 h of chilling, the chiller door was open, and the chiller wall was set to be absolutely insulated, and the air in the chiller was convectively transferred to the outside through the door. During the second phase of chilling from 8 to 14 h, the chiller door was closed, and the chiller wall was absolutely insulated. The air in the chiller exchanged heat only through the door. The outside temperature was 30 °C. The main material of the wall was cement and plastic board, and the convection heat transfer coefficient was set to 0.1.

Boundary treatment of the air outlet of the cooler [22]:

(1) Turbulent pulsating kinetic energy $\frac{\partial K}{\partial n}=0$;
(2) Pulsation energy dissipation $\frac{\partial \varepsilon }{\partial n}=0$;

*K* and *ε* are local unidirectional processing.

The commonly used grids in computational fluid dynamics (CFD) are structured or unstructured grids. Two meshing methods are used for numerical simulation. Chilling at 0 h was a structured grid, but the rest of chilling was an unstructured grid, and the dimensional accuracy was divided by 100 mm.

### 2.3. Microbiological Sampling in the Air and on the Surface of Pig Carcasses

At 0, 2, 4, 6, 8, 10, 12, and 14 h of chilling, microorganisms in the air were captured by the air plankton sampler (ZR-2050, Qingdao Zhongrui Intelligent Instrument Co., Ltd., Qingdao, China.) and plate count agar (PCA, Nanjing Rongshengda Experimental Instrument Co., Ltd., Nanjing, China). In each plate, 500 L of the air were captured. Due to the low abundance of microorganisms in the air, the air-borne microorganisms were enriched by incubating at 37 °C for 48 h. Then, the samples were suspended with 1 to 2 mL of sterile water and stored at −80 °C for further analyses. At each time point of a chilling day, three air samples at three plates were captured as replicates. Such a sampling procedure was repeated on 5 separate days. Thus, a total of 120 air samples were achieved for microbial sequencing.

At 2, 4, 6, 8, 10, 12, and 14 h of chilling, microorganisms on the surface of pig carcasses were obtained by swabbing the forelegs and the pleural cavity of pig carcasses with disposable sterile swabs (Yangsheng Medical, Yangzhou, Jiangsu, China). At each time point of a chilling day, two swabs were obtained from each of three carcasses. The sampling procedure was repeated on 5 separate days. Thus, a total of 210 swabs were obtained for sequencing. About 100 cm^2^ area of each part was swabbed. The swabs were stored at −80 °C for further use. The airborne microorganisms and the swabs containing microorganisms were sequenced.

### 2.4. 16S rRNA Sequencing

The airborne microorganisms and the swabbing microorganisms on the surface of pig carcasses were sent to Shanghai Lingen Biological Technology Co., Ltd. for sequencing analysis. Specific steps were as follows. Three replicate samples from the air and the swabs were merged and microbial DNA was extracted from samples using an EZNA DNA kit (Omega Bio-tek, Norcross, GA, USA) according to manufacturer’s protocols. The V4-V5 region of the bacterial 16S ribosomal RNA gene was amplified by PCR (95 °C for 2 min, followed by 25 cycles at 95 °C for 30 s, 55 °C for 30 s, 72 °C for 30 s, and a final extension at 72 °C for 5 min) using primers 515F: 5′-barcode-GTGCCAGCMGCCGCGG)-3′ and 806R 5′-GGACTACHVGGGTWTCTAAT-3′, where barcode is an eight-base sequence unique to each sample. PCR reactions were performed in triplicate in a mixture (20 μL) containing 4 μL of 5 × FastPfu buffer, 2 μL of 2.5 mM dNTPs, 0.8 μL of each primer (5 μM), 0.4 μL of FastPfu polymerase, and 10 ng of template DNA. Amplicons were extracted from 2% agarose gels and purified using AxyPrep DNA gel extraction kit (Axygen Biosciences, Union City, CA, USA) according to the manufacturer’s instructions.

Purified PCR products were quantified by Qubit^®^ 3.0 (Invitrogen, Waltham, MA, USA) and every twenty-four amplicons with different barcodes were mixed equally. The pooled DNA product was used to construct an Illumina pair-end library following Illumina’s genomic DNA library preparation procedure. Sequencing libraries were generated using NEB Next^®^ Ultra™ DNA Library Prep Kit for Illumina (NEB, Ipswich, MA, USA) following the manufacturer’s recommendations, and index codes were added. The library quality was assessed on the Qubit@ 2.0 Fluorometer (Thermo Scientific, Waltham, MA, USA) and Agilent Bioanalyzer 2100 system. Then, the amplicon library was paired-end sequenced (2 × 250) on an Illumina MiSeq platform (Shanghai BIOZERON Co., Ltd., Shanghai, China) according to the standard protocols.

### 2.5. Detection of Drug-Resistant Genes in Microbial Samples

Several resistant genes were selected according to the types of commonly used veterinary drugs. The names and primer sequences are listed in Table 1. All microbial samples were collected from the air, and the carcasses were used for detecting all selected resistant genes. The real-time quantitative PCR (qPCR) was performed with a commercial SYBR Premix Ex Taq II kit (Tli RNase H Plus, Baori Medical, Beijing, China). The total volume was 20 μL, containing 10 μL SYBR Premix Ex Taq II (TliRNase H Plus), 0.8 μL forward primers, 0.8 μL reverse primers, 0.4 μL ROX reference dye II, 6 μL ddH_2_O and 2 μL template DNA. A real-time fluorescence quantitative PCR instrument (QuantStudio 6, Thermo Fisher Scientific, Waltham, MA, USA) was used for quantification. The template DNA of each microbial sample was diluted to 5^−1^ gradient and was repeated three times. The PCR protocol was as follows: 30 s at 95 °C, followed by 40 cycles of 5 s at 95 °C, 34 s at 60 °C, and then, a final melting curve stage at 95 °C for 15 s, 60 °C for 1 min, and 95 °C for 15 s.

### 2.6. Data Analysis

Usearch (version 10, http://drive5.com/uparse/ (accessed on 26 November 2019)) was used to perform clustering analysis of operational taxonomic units (OTUs). Alpha diversity and community composition analysis were calculated by Mothur (version 1.30.1, http://www.mothur.org/wiki/Schloss_SOP#Alpha_diversity (accessed on 26 November 2019)) and R software.

The effects of sampling time and site on relative abundances of drug-resistant genes were evaluated by analysis of variance. The means were compared by Duncan’s multiple range test using the SAS program (version 8.1, SAS Institute Inc., Cary, NC, USA). The Graphpad Prism software (version 7.0, https://www.graphpad.com/ (accessed on 30 April 2020)) was applied to prepare images.

## 3. Results

### 3.1. Fluid Simulation Modeling for Chilling Process of Pig Carcasses

#### 3.1.1. 3D Modeling of a Chiller

**Figure 1:**
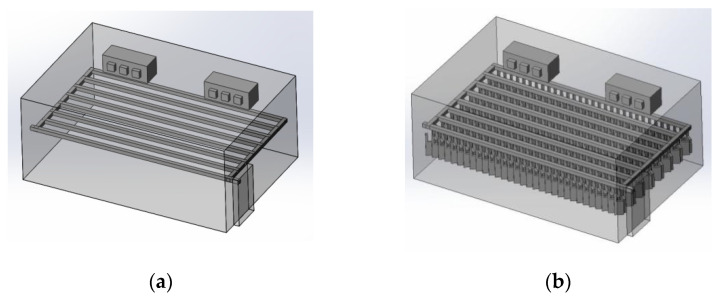
3D model of chiller and pig carcass after cooling for 0 h (**a**) and 8 h (**b**).

The premise of building a simulation model is to establish an intuitive and accurate 3D model. For the convenience of calculation, pig carcasses with an irregular shape were considered cuboid (including the legs). One half of a carcass was considered a semi-cuboid. The distance between two half carcasses on the same track was 0.1 m. The chiller was empty at 0 h of chilling, but it was full of pig carcasses at 8 h. The 3D models are shown in Figure 1.

#### 3.1.2. Simulating Changes of Temperature, Humidity, and Wind Speed and Grid Division

**Figure 2:**
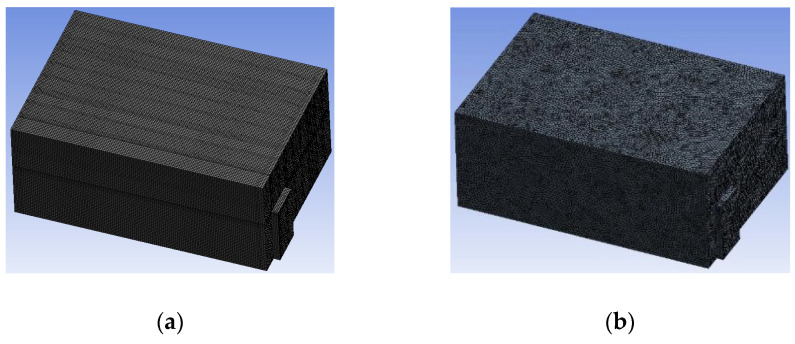
Grid division structure diagram of chiller after cooling for 0 h (**a**) and 8 h (**b**).

The wind speed at the outlet of the chiller was maintained at 15–20 m/s during carcass chilling. At 0 h, the structured mesh was suitable for a cold store according to standard geometry. At subsequent time points, pig carcasses were placed in the chiller and unstructured meshing was applied to make the simulation results more accurate. The real values of measured temperature and humidity and predicted meshing results are shown in Table 2. The meshing results are shown in Figure 2.

#### 3.1.3. Simulation Results

In order to visualize the changes in wind speed, temperature, and humidity of the chiller during chilling, the transverse diagrams were captured for the wind speed, temperature, and humidity in the chiller at 0, 2, 4, 6, 8, 10, 12, and 14 h. Each set of pictures contained six transverse views, from high to low positions of the chiller; the track surface; the upper, middle, and lower positions of pig carcasses; and the ground (Table 3).

Although the wind speed distribution was slightly different during chilling, no significant changes were observed, indicating that the arrangement of pig carcasses did not affect the distribution of airflow in the chiller. The wind speed was higher in the places where the fan was located or close to the ground. This was because the high-speed air flow blown by the fan was on the top layer, and the cold air was not spread upwards but downwards. When the overall wind speed in the chiller was high, the air exchange on the chiller door was not obvious. The airflow of the chiller had good symmetry in the horizontal direction. Thus, the layout of the chiller and the arrangement of pig carcasses are reasonable.

The temperature field within the first 8 h of chilling was significantly different from that from 10 to 14 h of chilling. Within the first 8 h, the overall temperature gradually increased, and the temperature close to the door was significantly higher than the inside parts of the chiller. The color of the chiller was red, indicating that heat exchange occurred at the door from the external hot air to the cold air in the chiller. This resulted in a temperature increase close to the door, and eventually the heat was radiated to a quarter of the chiller. In the second stage from 10 to 14 h, the temperature of the chiller declined rapidly, and the temperature field uniformly showed blue, indicating that the temperature was low and evenly distributed. Because the chiller door was closed after the chiller was filled with pig carcasses, the heat exchange with the hot air outside the door was stopped, and the circulating refrigeration in the chiller was no longer interfered with by external heat. However, the color of the wall near the door was still slightly “red shifted”, indicating that the temperature here was higher than other parts of the chiller. This may be because the chiller wall was not completely insulated, and the external heat could still penetrate through the wall.

The humidity change was similar to temperature. In the first stage from 0 to 8 h, the overall humidity gradually increased, and the humidity close to the door of the chiller was significantly higher than the inside parts of the chiller. The red color of the chiller door indicated that the heat exchange between the inside and outside of the chiller increased the air humidity. During this period, the humidity field was more unevenly distributed than the temperature field, indicating that the heat exchange had a higher influence on the humidity in the chiller than the temperature. In the second stage from 10 to 14 h, the humidity in the chiller declined rapidly, and the humidity field inside the chiller was evenly distributed. This was because the chiller door had been closed, and the heat exchange with the hot air outside the door was stopped. Contrary to the distribution of the temperature field, the color of the wall near the chiller door was slightly “blue shifted”, which indicates that the humidity here was slightly lower than the overall humidity in the chiller.

### 3.2. The Microbiota Composition in the Air and on the Surface of Pig Carcasses Changed Greatly during Chilling

#### 3.2.1. Diversity of the Microbiota

**Figure 3:**
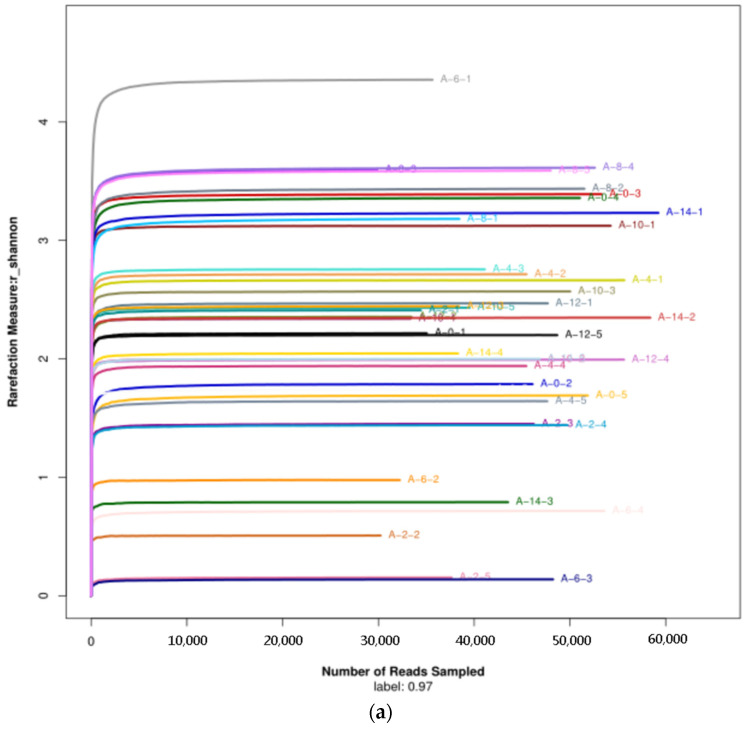

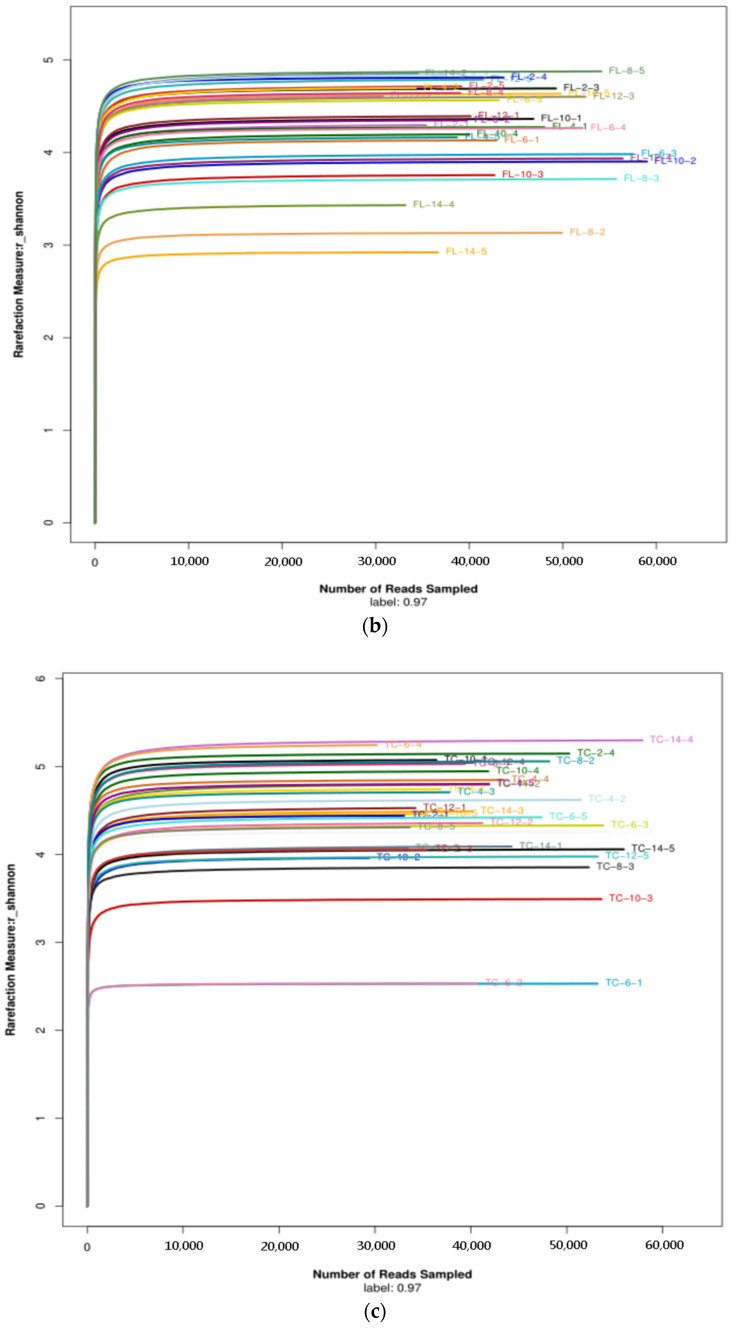
Shannon–Wiener curves of air (**a**), foreleg (**b**), and thorax (**c**) microbial samples throughout the cooling process.

The Shannon–Wiener curves of the microbial samples from the air, the surface of forelegs, and pleural cavity of pig carcasses tended to be flat, indicating that sequencing data can well reflect the microbial information in samples (Figure 3).

**Figure 4:**
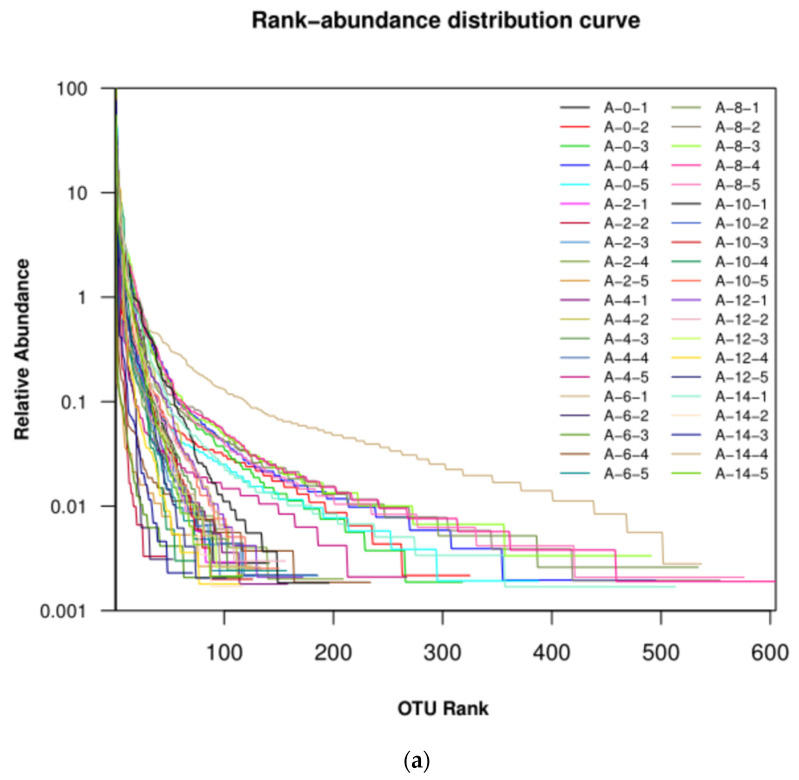

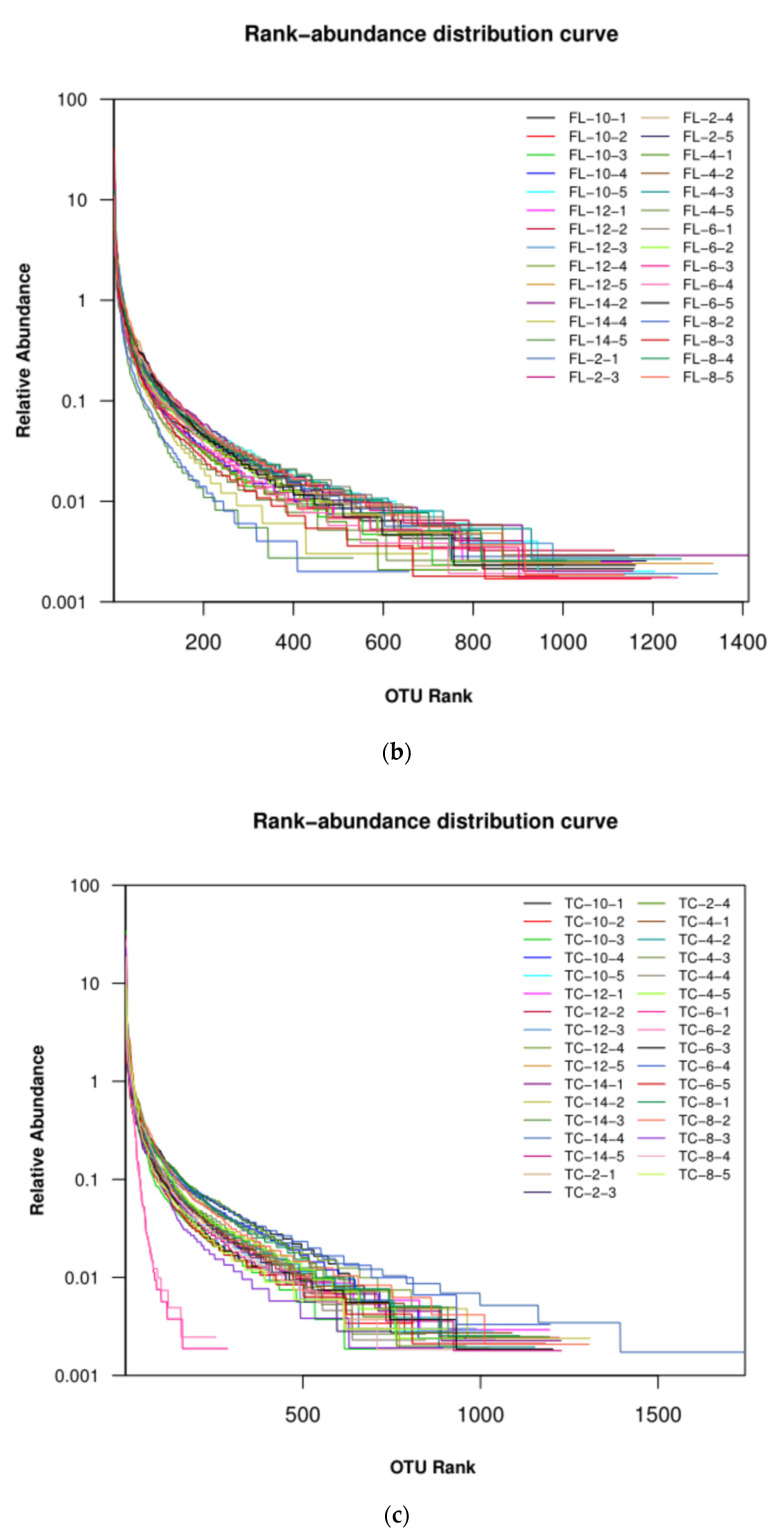
Rank-abundance curves of air (**a**), foreleg (**b**), and thorax (**c**) microorganisms throughout the cooling process.

Rank-abundance curves reflect species abundance and uniformity. The width of a curve reflects the abundance of the species. The larger the curve spans on the horizontal axis, the higher the abundance of the species. The microbial samples from the surface of the pig carcass were more abundant than those in the air (Figure 4). The smoothness of a curve reflects the evenness of the microbial species in the samples. The smoother the curve, the more uniform the species distribution, and the steeper the curve, the more uneven the species distribution. The uniformity of the microorganism samples from the surface of the pig carcasses was better than those in the air.

**Figure 5:**
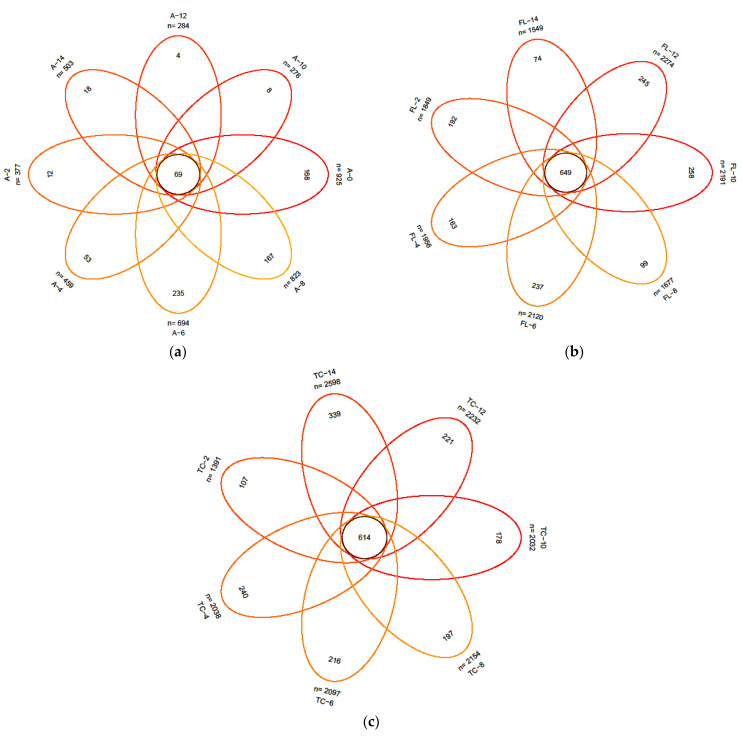
Venn diagrams of air (**a**), foreleg (**b**), and thorax (**c**) microorganisms throughout the cooling process.

A Venn diagram showed that the OTUs in the microbial samples from the air and the surface of forelegs and pleural cavity varied with chilling time (Figure 5). When comparing the number of OTUs between air samples and swabs, we observed much smaller numbers of OTUs in air-borne samples. This is due to the loss of non-cultivable microorganisms. The number of OTUs in the air samples was the smallest at 10 h and the greatest at 0 h (276 vs. 925). The number of OTUs on the surface of the forelegs was the smallest at 14 h and the greatest at 12 h (1549 vs. 2274). The number of OTUs from the surface of the pleural cavity was the smallest at 2 h and the greatest at 14 h (1391 vs. 2598).

#### 3.2.2. The Alterations in Microbiota Composition during Chilling

**Figure 6:**
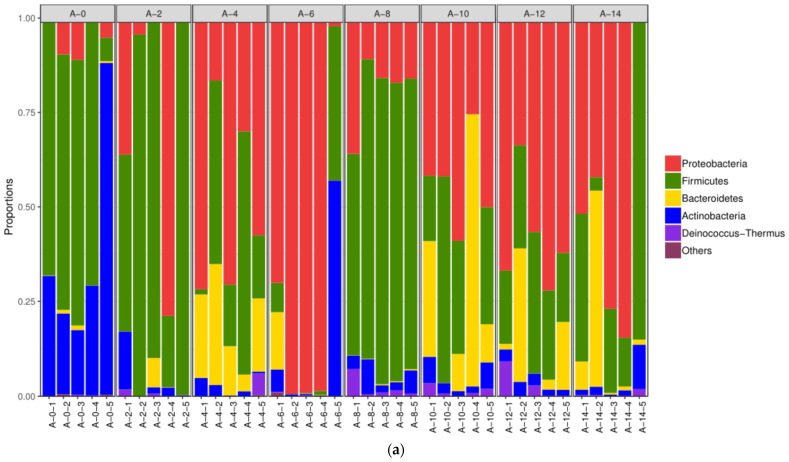

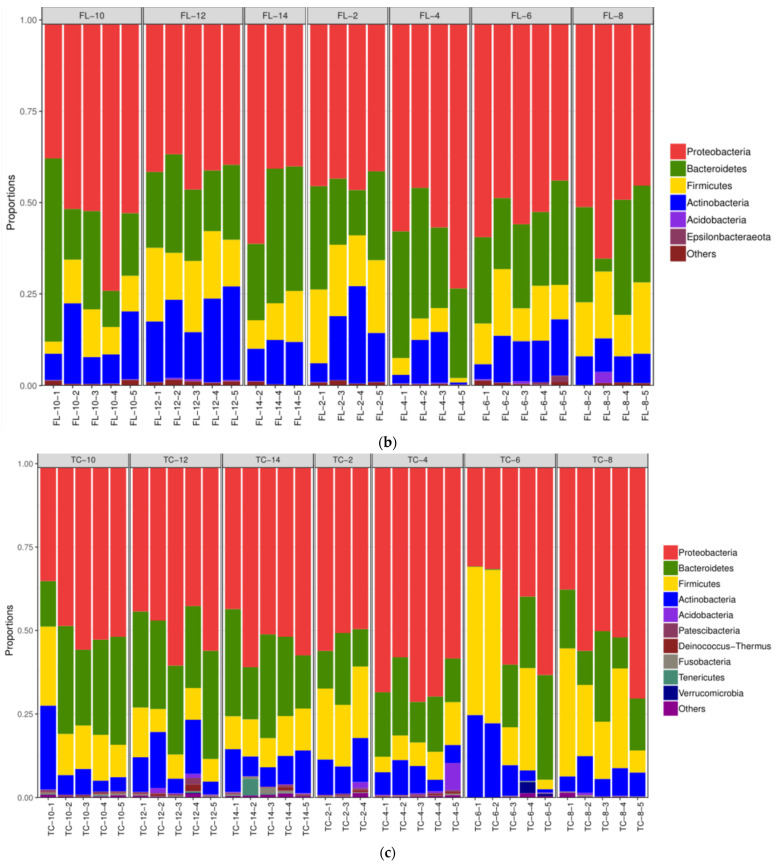
Community structure components of air (**a**), foreleg (**b**), and thorax (**c**) microorganisms at the phylum level.

At the phylum level, Proteobacteria, Bacteroides, Firmicutes, and Actinobacteria were the dominant bacteria in the airborne samples. At 0 h, the relative abundance of Firmicutes was the highest for the first four merged air-borne samples (Figure 6a). However, in the fifth merged sample, the abundance of Actinobacteria was the highest. At 2 h and subsequent time points when pig carcasses and workers went in and out, the dominant phyla changed greatly. The relative abundance of Proteobacteria and Bacteroides increased substantially, and the fifth merged samples showed a big difference from other samples at most time points. Such changes could be associated with practices and hygiene management in the factory. The dominant phyla on the surface of the forelegs were Proteobacteria, Bacteroides, Firmicutes, and Actinomycetes (Figure 6b). Although some variations exist in the relative abundance of these phyla, the phylum composition kept relatively constant during chilling. Proteobacteria, Bacteroides, Firmicutes, and Actinomycetes were predominant on the pleural cavity of pig carcasses and the relative abundance of Proteobacteria was also the highest (Figure 6c). As noted above, the microbial composition showed a great difference between the air-borne samples and swabs because some air-borne microorganisms could not be cultivated.

**Figure 7:**
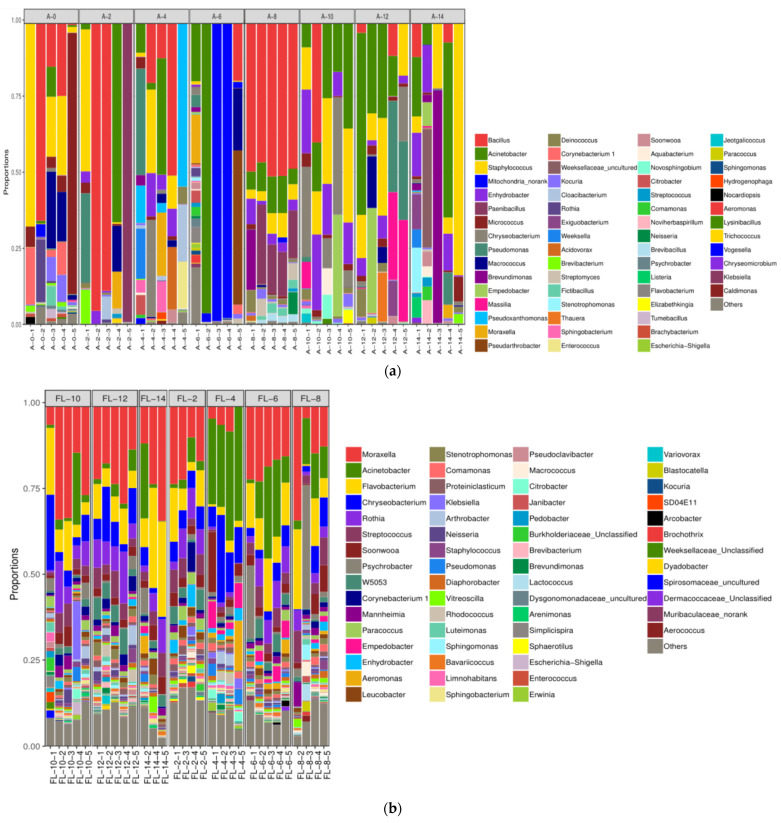

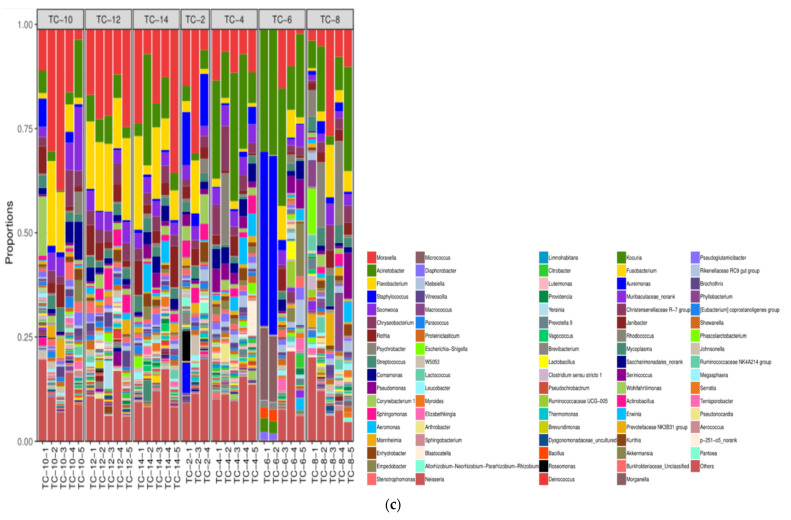
Community structure components of air (**a**), foreleg (**b**), and thorax (**c**) microorganisms at the genus level.

At the genus level, there were greater than seventy genera in the air and on the surface of pig carcasses; however, the microbial composition was quite different (Figure 7). In the air, Bacillus, Acinetobacter, and Staphylococcus were the dominant genera, and the genera varied greatly with time during chilling (Figure 7a). It is difficult to find a distinct trend. This could be due to the complexity of microenvironment (e.g., temperature and humidity) and movement of workers and pig carcasses. On the surface of the forelegs, Moraxella, Acinetobacter, and Flavobacterium were the main genera (Figure 7b). Moraxella was the predominant genus through the period of chilling. The relative abundance of Flavobacterium increased greatly during chilling. On the pleural cavity, Moraxella, Acinetobacter, and Flavobacterium were the predominant genera (Figure 7c). The abundance of Acinetobacter showed an obvious increase, but the abundance of Moraxella declined as chilling time increased, indicating that Acinetobacter may be resistant to low temperature but not for Moraxella.

#### 3.2.3. The Difference in the Microbiota Composition among Sampling Places

**Figure 8:**
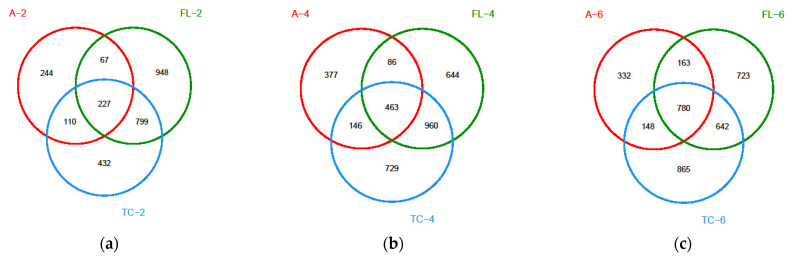

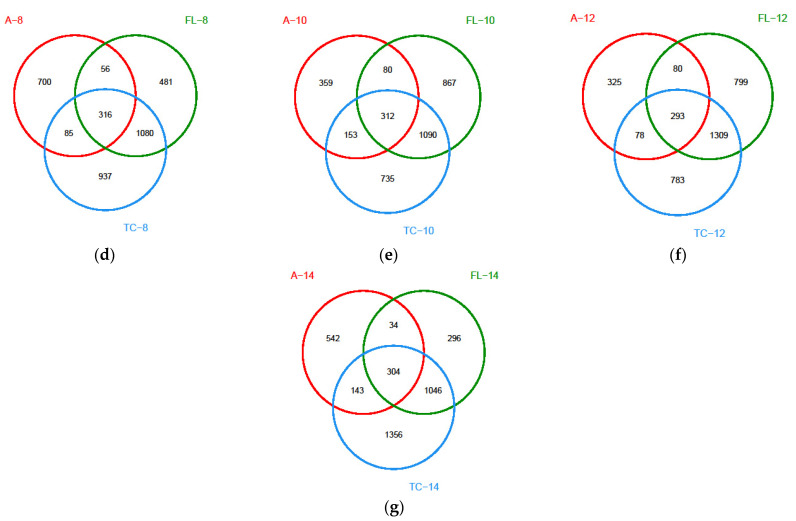
Venn diagrams of microorganisms at three locations (“A” in the figure represented air microorganisms, “FL” represented forelegs microorganisms, and “TC” represented thoracic microorganisms) during the same cooling time ((**a**–**g**) were cooling for 2 h, 4 h, 6 h, 8 h, 10 h, 12 h, and 14 h, respectively).

Venn diagrams showed differences in the microbiota composition among microbial samples from the air and the surface of forelegs and pleural cavity (Figure 8a–g). The numbers of overlapping OTUs for the three groups were the greatest at 6 h and the smallest at 2 h (780 vs. 227). The numbers of overlapping OTUs for the pleural cavity or the surface of foreleg and the air were also observed the greatest at 6 h and smallest at 2 h (943 vs. 294 for pleural cavity and 928 vs. 337 for foreleg). The OTUs on the surface of forelegs showed a great similarity to those on the pleural cavity, indicating that the microbiota composition on the surface of pig carcasses tended to be consistent.

### 3.3. Drug-Resistant Genes in Microbiota Samples from the Air and the Surface of Pleural Cavity and Foreleg

#### 3.3.1. Effect on the Number of Drug-Resistant Genes

It is a challenge to extract lowly abundant microbial genes for detecting antibiotic resistant genes. To minimize the error, all merged samples at each time point were tested in three replicates, and only samples with at least two positive results were considered resistance positive. As shown in Table 4, six resistant genes, that is, sul Ⅰ and sul Ⅱ (resistant to sulfonamides), qepA (resistant to quinolones), blaCTX-M-2 (resistant to β-lactams), and tetA and tetC (resistant to tetracycline) were detectable in all airborne samples across the chilling time points. The blaTEM (resistant to β-lactams), ermA (resistant to erythromycins), tetM (resistant to tetracycline), aac (6′)-Ib-cr and qnrA (resistant to quinolones) were also detectable by 75% to 87.5% of airborne samples. In addition, blaSHV (resistant to β-lactams), ermB (resistant to erythromycins), and ermC (resistant to erythromycins) were detectable in low abundance. Of the 14 selected drug-resistant genes, 11, 11, 10, 12, 10, 12, 11 and 6 genes were detected at 0, 2, 4, 6, 8, 10, 12, and 14 h, reflecting the alterations in the microbiota composition.

On the surface of the pleural cavity, sul Ⅰ, sul Ⅱ, qepA, blaCTX-M-2, tetA, tetC, tetM, and qnrA were detectable in all samples. The blaTEM, ermA, aac (6′)-Ib-cr, and blaSHV were also detectable by percentages of 57.1% to 87.5%. The ermB and ermC were not detected. Eleven, 12, 12, 10, 11, 11, and 10 of target genes were detected at 2, 4, 6, 8, 10, 12, and 14 h, respectively.

On the surface of the forelegs, only five genes, namely, sul Ⅰ, sul Ⅱ, blaCTX-M-2, tetA, and tetC, were detectable in all samples. The ermA, tetM, qnrA, blaTEM, qepA, aac (6′)-Ib-cr, and ermB genes were detectable by a percentage varying from 28.6% to 71.4%. However, blaSHV and ermC were not detectable. As chilling time extended, the detectable drug-resistant genes increased from six at 2 h to 12 at 8 h and then decreased to five at 14 h.

#### 3.3.2. Effect on the Abundance of Drug-Resistant Genes

In the airborne samples, the relative abundance of blaTEM, ermA, tetA, tetC, and tetM was higher than that of sul Ⅱ, aac (6′)-Ib-cr, sul Ⅰ, blaSHV, qepA, blaCTX-M-2, and qnrA (*p* < 0.05, Table 5). As chilling time increased, the relative abundance of sul Ⅰ and tetM decreased, while the abundance of blaSHV increased. The other genes did not show a single trend.

In the pleural cavity, the relative abundance of all the detected genes did not differ at 2 h (*p* > 0.05, Table 5), but significant differences were observed in the remaining chilling time points (*p* < 0.05). The relative abundance of ermA, tetA, and sul Ⅱ was high, while that of blaTEM, qepA, and blaCTX-M-2 was low. Thus, erythromycin, sulfa, and tetracycline had a greater impact on the microbes on the surface of the pleural cavity of pig carcasses.

On the surface of the forelegs, there was no significant difference in relative abundance of the detected drug-resistant genes at 2 h (*p* > 0.05, Table 5), but significant differences were observed at the subsequent time points (*p* < 0.05). The relative abundance of sul Ⅰ, sul Ⅱ, and tetA was high, while it was low for blaTEM, ermB, tetC, and qnrA.

## 4. Discussion

Temperature and humidity have been shown to be important for restricting the growth of microorganisms. In a close chiller, the temperature and humidity fields were actually not uniform. The cold flow from air conditioners was roughly divided into two ways, one directed to the inside of the chiller and the other directed to the outside of the chiller. Such a difference in wind speed, temperature, and relative humidity may result in different evaporative loss of pig carcasses [7]. Usually, high wind speed with high relative humidity will cause low evaporative loss. During chilling, the cold flow from the air conditioner goes down under gravity, and forelegs close to the ground could get more cold air. Thus, the lower plane in a chiller is relatively evenly distributed in wind speed, temperature, and humidity. The middle plane had the lowest wind speed but the highest temperature. During the whole chilling, the humidity distribution was relatively uniform, which may have resulted from the use of circulating refrigeration in the chiller and the relatively single source of moisture.

The wind speed distribution in the chiller was uneven, which may be related to the arrangement of pig carcasses and the external air circulation. The unevenly distributed wind speed and temperature fields in the chiller may cause different cooling rates of pig carcasses at different locations. Due to the difference in position, the cooling rate of different parts of the same pig carcass was not synchronized.

We found that during chilling, when the temperature and humidity gradually increased (within the first 8 h), Acinetobacter dominated the microbes on the surface of the front legs, and when the temperature and humidity decreased between 10 and 14 h, Moraxella dominated the microorganisms. In terms of drug-resistant genes, when the temperature and humidity were high, the detection rate and relative quantitative values of drug-resistant genes were higher than those at 14 h when the temperature and humidity were the lowest. This indicates that changes in temperature and humidity during the chilling process could lead to the differences in the predominant species of microorganisms and the results of drug-resistant genes on the surface of pig carcasses.

Studies have shown that the dominant spoilage bacteria in chilled pork were Pseudomonas, Enterobacteriaceae, Thermomycetes, Lactobacillus, Moraxella, and Acinetobacter [24,25]. The present study confirmed these observations. The microbiota on the surface of pig carcasses may be derived from different sources, in particular, the workers’ hands. Many skin-residing bacteria were detected in the air of the chiller and on the surface of the pig carcasses. This could be attributed to contamination from the workers who handled pig carcasses.

Among the 52 bacterial phyla currently recognized on the earth, five to seven phyla are known to exist in the gastrointestinal tract of mammals. Firmicutes and Bacteroidetes are dominant in the gut, while Actinomycetes are less abundant [26]. In this study, we observed that Bacteroidetes are predominant on the surface of pig carcasses, indicating that pig carcasses may be contaminated by animal feces before slaughter or by destroyed gut by mishandling. In addition to exogenous enteropathogenic Proteus, healthy mammalian intestines also contain some symbiotic flora belonging to Proteus as their natural intestinal flora [27,28]. Among the four main phyla of intestinal flora (Firmicutes, Bacteroidetes, Proteus, and Actinomycetes), Proteus is the most unstable over time [29]. In this study, the pig carcass surface microorganisms contained the above four phyla, and all of them were dominant bacteria. Therefore, it can be explained that the original intestinal microorganisms are one of the main sources of pig carcass surface microorganisms. In addition to the above main sources, Actinomycetes are also widely distributed in soil and water, indicating that the microorganisms on the surface of pig carcass may come from the soil attached to the surface of pig carcass not completely washed and the cooling water for washing before chilling.

The sequencing results showed that at the phylum level, the bacteria on the surface of the pig carcass were more abundant than those in air. This could be because some microbial genes of non-cultivable microorganisms have been lost during plate cultivation. On the other hand, multiple contamination still exists during slaughtering handling, even if decontamination operations are applied.

Animal manure in farms is considered to be the main reservoirs of drug-resistant genes, because a large number of resistant genes are often detected in these environments [15,16,30,31,32,33]. The tetA, tetB, and tetM genes have been frequently detected in livestock farms [34,35]. Chee-Sanford et al. [36] identified eight genes (tetO, tetQ, tetW, tetM, tetB (P), tetS, tetT, and otrA) from two swine lagoons and the subsurface soil. Tetracycline (tetO and tetW) and sulfonamide (sul I and sul II) resistant genes were found to be highly abundant in cattle, pigs, and chicken lagoons [37]. Therefore, the animal gut and the unwashed soil on the surface of pigs may be the origin of the drug-resistant genes from the surface of pig carcasses.

The abundance of drug-resistant genes was usually related to the use of these antibiotics in animal farms [16,38,39]. In the present study, the abundance of blaTEM, ermA, tetA, tetC, and tetM in airborne microorganisms was higher, while ermA, tetA, sul Ⅰ, and sul Ⅱ were more abundant in the surface of pig carcasses. This could be related to the abuse of β-lactam, sulfonamide, and tetracycline in pig farms. They may spread to humans through the meat chain. Therefore, reasonable or limited use of antibiotics, the establishment of corresponding scientific monitoring, and management systems in animal husbandry are important for limiting the adverse effects of antibiotic abuse and ensuring food safety.

## 5. Conclusions

There was a large difference in wind speed varying with locations, and the wind speed of the whole chiller was unevenly distributed. The wind speed at the air outlet of the air conditioner was the greatest, and it decreased rapidly along the wind path, with the smallest value at the center of the chiller. During chilling within the first 8 h, the temperature and humidity on the side close to the chiller door were higher than the inner side of the chiller. Then, the temperature and humidity distribution became more uniform. During chilling, microbial composition in the air and on the carcass surface were different. Fourteen drug-resistant genes were selected to identify in the air and on the surface of pig carcasses. These findings provide a new insight into the source of meat microorganisms and food safety control.

## Figures and Tables

**Table 1:** The primer sequences of selected drug-resistant genes.

Gene	Forward	Reverse
16S rRNA	GCTCGTGTCGTGAGATGTT	TGTAGCCCAGGTCATAAGG
sul Ⅰ	CACCGGAAACATCGCTGCA	AAGTTCCGCCGCAAGGCT
sul Ⅱ	CTCCGATGGAGGCCGGTAT	GGGAATGCCATCTGCCTTGA
blaSHV	AGCCGCTTGAGCAAATTAAAC	ATCCCGCAGATAAATCACCAC
blaTEM	CATTTCCGTGTCGCCCTTATTC	CGTTCATCCATAGTTGCCTGAC
ermA	AAGCGGTAAAACCCCTCGAG	TCAAAGCCTGTCGGATTGG
ermB	GAAAAGGTACTCAACCAAATA	CATTTGTTAAATTCATGGCAATGA
ermC	TCAAAACATAATATAGATAAA	GCTAATATTGTTTAAATCGTCAAT
qepA	AACTGCTTGAGCCCGTAGAT	GTCTACGCCATGGACCTCAC
blaCTX-M-2	CGTTAACGGCACGATGAC	CGATATCGTTGGTGGTRCCAT
aac(6′)-Ib-cr	TTGCGATGCTCTATGAGTGGCTA	CTCGAATGCCTGGCGTGTTT
tetA	GCTACATCCTGCTTGCCTTC	CATAGATCGCCGTGAAGAGG
tetC	CTTGAGAGCCTTCAACCCAG	ATGGTCGTCATCTACCTGCC
tetM	GTGGACAAAGGTACAACGAG	CGGTAAAGTTCGTCACACAC
qnrA	AGAGGATTTCTCACGCCAGG	TGCCAGGCACAGATCTTGAC

**Table 2:** Measured values of temperature and humidity at the outlet and door and grid division of the chiller.

Chilling Time/h	Outlet Temperature/°C	Outlet Humidity/%	Door Temperature/°C	Door Humidity/%	Number of Grids/Piece	Grid Type
0 h	8	97	10	98	786,360	Structured
2 h	10	94	12	99	1,848,737	Unstructured
4 h	11	97	12	99	1,934,154	Unstructured
6 h	12	98	13	100	2,424,042	Unstructured
8 h	12	98	13	100	2,638,668	Unstructured
10 h	10	86	10	86	2,638,668	Unstructured
12 h	6	83	6	83	2,638,668	Unstructured
14 h	3	74	3	74	2,638,668	Unstructured

**Table 3:** Fluid simulation of wind speed, temperature, and humidity in a chiller.

Time/h	Wind Speed (Air flow)	Temperature	Humidity
0 h	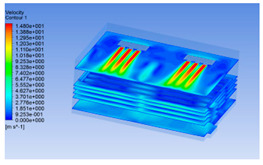	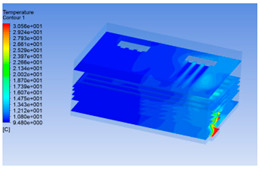	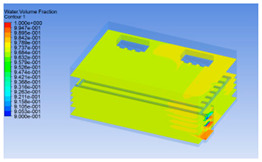
2 h	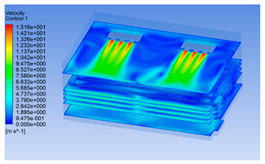	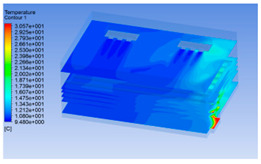	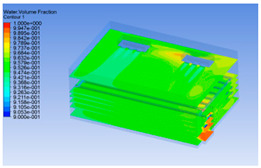
4 h	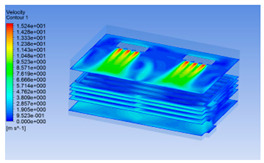	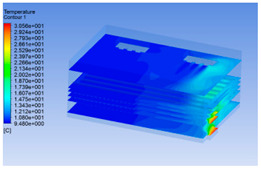	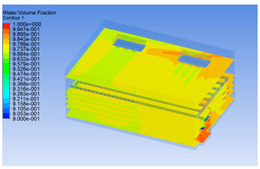
6 h	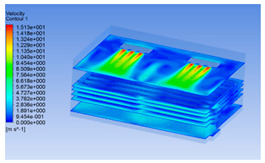	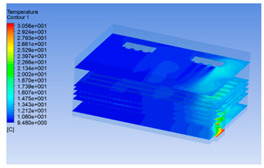	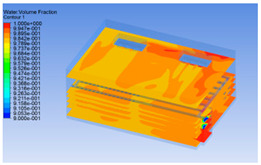
8 h	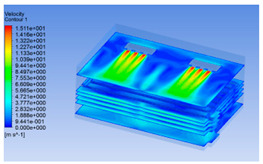	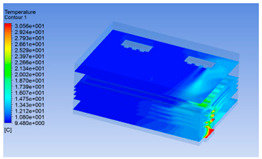	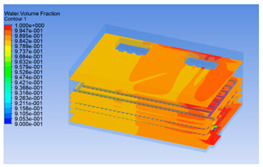
10 h	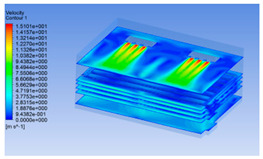	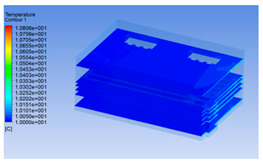	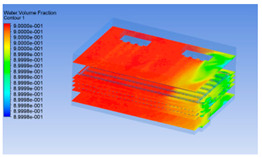
12 h	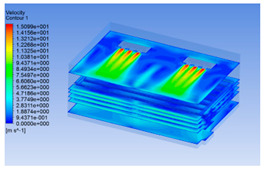	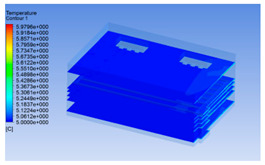	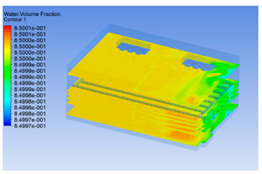
14 h	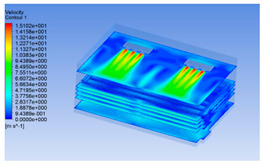	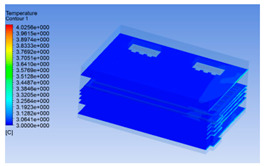	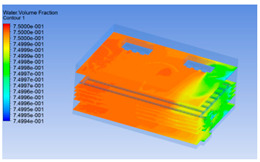

In the wind speed diagrams, different colors represent different wind speeds. The more biased the red color is, the higher the wind speed, and the more biased the blue, the lower the wind speed. Different colors represent different temperatures, and the more biased the red color iss, the higher the temperature, and the more biased the blue, the lower the temperature. The more biased the red, the higher the humidity, and the more biased the blue, the lower the humidity.

**Table 4:** Incidence of drug-resistant genes in microbiota samples from the air and the surface of pleural cavity and foreleg.

Locations	Time/h	sul Ⅰ	sul Ⅱ	blaSHV	blaTEM	ermA	ermB	ermC	qepA	blaCTX-M-2	aac(6′)-Ib-cr	tetA	tetC	tetM	qnrA
Airborne	0 h	+/+/+	+/+/+	-/+/-	+/+/+	-/+/+	-/-/-	-/-/-	+/+/+	-/+/+	+/-/+	+/+/+	+/+/-	-/+/+	+/-/+
	2 h	+/+/+	+/+/+	+/+/-	+/+/+	+/+/+	-/-/-	-/-/-	+/+/+	+/+/+	+/+/-	+/+/+	+/+/-	-/-/+	+/+/+
	4 h	+/+/+	+/+/-	-/-/-	+/+/+	+/+/+	-/-/-	-/-/-	+/+/+	+/+/+	-/-/-	+/+/+	+/+/+	+/-/+	+/+/+
	6 h	+/+/+	+/+/+	-/+/+	+/+/+	+/+/+	-/-/-	-/-/-	+/+/+	+/+/+	+/+/+	+/+/+	+/+/+	-/+/+	+/+/+
	8 h	+/+/+	+/+/+	-/+/-	+/+/+	-/-/+	-/+/-	-/-/-	+/+/+	+/+/+	+/+/+	+/+/+	+/+/+	-/+/+	+/+/+
	10 h	+/+/+	+/+/+	-/+/+	+/+/+	+/+/+	-/-/+	-/-/-	+/+/+	+/+/+	-/+/+	+/+/+	+/+/+	+/+/+	+/+/+
	12 h	+/+/+	+/+/+	-/-/-	-/+/+	+/+/+	-/-/+	-/-/-	+/+/+	+/+/+	+/+/+	+/+/+	-/+/+	-/+/+	+/+/+
	14 h	+/+/+	+/+/+	-/+/-	-/+/-	-/-/-	-/+/-	-/-/-	+/+/+	+/+/+	-/+/-	+/+/+	+/+/-	-/+/-	+/-/-
Pleural cavity	2 h	+/+/+	+/+/+	+/+/+	+/-/-	+/+/+	-/-/+	-/-/-	+/+/-	-/+/+	+/+/+	+/+/+	+/+/+	+/+/+	+/+/-
	4 h	+/+/+	+/+/+	+/+/-	+/+/+	+/+/+	-/+/-	-/-/-	+/+/+	+/+/+	+/+/-	+/+/+	+/+/+	-/+/+	+/+/+
	6 h	+/+/+	+/+/+	+/+/-	+/+/+	+/-/+	-/-/-	-/-/-	+/+/+	+/+/+	+/+/+	+/+/+	+/+/-	+/+/-	+/+/+
	8 h	+/+/+	+/+/+	+/-/-	+/+/+	+/+/+	-/-/-	-/-/-	+/+/+	+/+/+	-/+/-	+/+/+	+/+/+	+/+/+	+/+/+
	10 h	+/+/+	+/+/+	-/+/+	+/+/+	-/-/+	+/-/-	-/-/-	+/+/+	+/+/+	+/+/-	+/+/+	+/+/+	+/-/+	+/+/+
	12 h	+/+/+	+/+/+	-/+/-	-/+/+	+/+/+	-/+/-	-/-/-	+/+/+	+/+/+	+/+/-	+/+/+	+/+/+	+/+/+	+/+/+
	14 h	+/+/+	+/+/+	+/-/-	+/+/+	+/+/+	-/-/-	-/-/-	+/-/+	+/+/+	-/-/+	+/+/+	+/+/+	+/+/+	+/-/+
Foreleg	2 h	+/+/+	+/+/+	-/-/-	-/-/+	-/-/+	-/-/+	-/-/-	-/-/-	+/-/+	+/+/+	+/+/+	-/+/+	-/-/-	+/-/-
	4 h	+/+/+	+/+/+	-/+/-	-/+/+	+/+/+	-/-/-	-/-/-	+/+/+	+/+/+	-/-/-	+/+/+	+/-/+	+/-/+	+/+/+
	6 h	+/+/+	+/+/+	-/-/-	+/+/+	+/+/+	-/-/-	-/-/-	+/+/+	+/+/+	-/+/+	+/+/+	+/+/+	+/+/+	-/+/+
	8 h	+/+/+	+/+/+	-/-/-	+/+/+	+/-/+	+/+/+	-/-/-	+/+/+	+/-/+	+/-/+	+/+/+	+/+/+	+/+/+	+/+/+
	10 h	+/+/+	+/+/+	-/-/+	-/+/+	+/+/-	-/+/-	-/-/-	+/-/+	+/+/+	-/-/-	+/+/+	+/+/+	+/+/+	+/+/+
	12 h	+/+/+	+/+/+	+/-/-	-/+/-	+/+/+	-/+/+	-/-/-	-/+/-	+/+/+	+/+/-	+/+/+	+/+/+	+/-/+	+/+/-
	14 h	+/+/+	+/+/+	-/+/-	-/-/-	+/-/-	-/-/+	-/-/-	+/-/-	+/+/+	-/-/-	+/+/+	+/+/+	+/-/-	-/-/+

Note: “+”, detected; “-” not detected.

**Table 5:** Relative abundance of drug-resistant genes in microbiota samples from the air and the surface of pleural cavity and foreleg.

Locations	Time/h	sul Ⅰ	sul Ⅱ	blaSHV	blaTEM	ermA	qepA	blaCTX-M-2	aac(6′)-Ib-cr	tetA	tetC	tetM	qnrA
airborne	0 h	1.000 ^Aa^	1.000 ^Aa^	—	1.000 ^Ba^	1.000 ^Ca^	1.000 ^Aa^	1.000 ^Aa^	1.000 ^Aa^	1.000 ^Ba^	1.000 ^Aa^	1.000 ^Aa^	1.000 ^Aa^
	2 h	0.001 ^Eg^	0.002 ^Ff^	0.001 ^Cg^	0.029 ^Ec^	0.394 ^Ea^	0.000 ^Ch^	0.001 ^Eg^	0.002 ^Df^	0.021 ^Fd^	0.047 ^Db^	—	0.008 ^Ee^
	4 h	0.360 ^Ce^	0.025 ^Cj^	—	1.261 ^Ac^	3.228 ^Ab^	0.110 ^Bh^	0.076 ^Bi^	—	4.736 ^Aa^	0.375 ^Bd^	0.211 ^Bf^	0.189 ^Bg^
	6 h	0.017 ^Dg^	0.002 ^Fj^	0.004 ^Bi^	0.119 ^Dc^	0.465 ^Da^	0.000 ^Ck^	0.013 ^Dh^	0.027 ^Be^	0.033 ^Ed^	0.171 ^Cb^	0.022 ^Cf^	0.022 ^Df^
	8 h	0.000 ^Fd^	0.105 ^Ba^	—	0.005 ^Gb^	—	0.000 ^Cd^	0.000 ^Fd^	0.000 ^Fd^	0.000 ^Hd^	0.000 ^Hd^	0.000 ^Ed^	0.004 ^Fc^
	10 h	0.391 ^Bc^	0.011 ^Dj^	0.006 ^Ak^	0.948 ^Cb^	2.415 ^Ba^	0.000 ^Cl^	0.020 ^Cg^	0.025 ^Cf^	0.181 ^Cd^	0.013 ^Eh^	0.012 ^Di^	0.085 ^Ce^
	12 h	0.001 ^Eg^	0.010 ^Ec^	—	0.028 ^Fa^	0.025 ^Fb^	0.000 ^Ch^	0.001 ^Eg^	0.001 ^Eg^	0.008 ^Gd^	0.005 ^Fe^	0.000 ^Eh^	0.002 ^Gf^
	14 h	0.000 ^Fd^	0.025 ^Cb^	—	—	—	0.000 ^Cd^	0.001 ^Ec^	—	0.034 ^Da^	0.001 ^Gc^	—	—
Pleural cavity	2 h	1.000 ^Ba^	1.000 ^Fa^	1.000 ^Aa^	—	1.000 ^Da^	1.000 ^Ba^	1.000 ^Aa^	1.000 ^Ba^	1.000 ^Fa^	1.000 ^Ba^	1.000 ^Ba^	1.000 ^Ca^
	4 h	0.636 ^Ci^	3.283 ^Ad^	0.879 ^Bh^	0.328 ^Al^	19.569 ^Aa^	0.600 ^Cj^	0.511 ^Bk^	2.333 ^Ae^	15.653 ^Ab^	12.966 ^Ac^	1.293 ^Ag^	1.761 ^Bf^
	6 h	0.087 ^Fa^	0.000 ^Gc^	0.000 ^Dc^	0.000 ^Fc^	0.000 ^Fc^	0.000 ^Gc^	0.001 ^Gb^	0.000 ^Ec^	0.001 ^Gb^	0.000 ^Gc^	0.000 ^Gc^	0.001 ^Gb^
	8 h	0.030 ^Gh^	1.716 ^Db^	—	0.022 ^Ei^	0.148 ^Ed^	0.057 ^Df^	0.050 ^Fg^	—	8.528 ^Ba^	0.016 ^Fj^	0.125 ^De^	0.190 ^Ec^
	10 h	1.162 ^Ad^	1.874 ^Bc^	0.131 ^Ch^	0.260 ^Bf^	—	1.102 ^Ae^	0.164 ^Dg^	0.120 ^Di^	2.939 ^Ca^	0.049 ^Ek^	0.075 ^Fj^	2.304 ^Ab^
	12 h	0.323 ^De^	1.522 ^Ec^	—	0.209 ^Cf^	1.847 ^Cb^	0.046 ^Ek^	0.108 ^Eh^	0.158 ^Cg^	1.882 ^Ea^	0.060 ^Dj^	0.092 ^Ei^	0.944 ^Dd^
	14 h	0.211 ^Ee^	1.821 ^Cc^	—	0.068 ^Di^	5.890 ^Ba^	0.011 ^Fj^	0.207 ^Cf^	—	2.396 ^Db^	0.156 ^Ch^	0.313 ^Cd^	0.159 ^Fg^
Foreleg	2 h	1.000 ^Fa^	1.000 ^Ga^	—	—	—	—	1.000 ^Aa^	1.000 ^Aa^	1.000 ^Ea^	1.000 ^Aa^	—	—
	4 h	1.341 ^Cc^	5.474 ^Fa^	—	0.051 ^Ag^	0.216 ^Ae^	—	0.453 ^Cd^	—	2.674 ^Bb^	0.008 ^Bh^	—	0.085 ^Af^
	6 h	1.131 ^Db^	12.416 ^Ba^	—	0.009 ^Ch^	0.160 ^Cd^	—	0.087 ^Ee^	0.018 ^Cf^	0.922 ^Fc^	0.000 ^Di^	—	0.011 ^Cg^
	8 h	0.559 ^Gc^	7.694 ^Da^	—	0.001 ^Df^	0.003 ^Ed^	—	0.001 ^Gf^	0.001 ^Df^	0.675 ^Gb^	0.000 ^Dg^	—	0.002 ^Ee^
	10 h	1.016 ^Ec^	14.536 ^Aa^	—	0.014 ^Bf^	0.178 ^Bd^	—	0.051 ^Fe^	—	1.856 ^Cb^	0.000 ^Dh^	—	0.006 ^Dg^
	12 h	3.631 ^Ab^	9.891 ^Ca^	—	—	0.126 ^Df^	—	0.616 ^Bd^	0.146 ^Be^	1.240 ^Dc^	0.001 ^Ch^	—	0.024 ^Bg^
	14 h	1.954 ^Bc^	6.620 ^Ea^	—	—	—	—	0.115 ^Dd^	—	3.131 ^Ab^	0.000 ^De^	—	—

Note: Different uppercase letters in the same column indicate significant differences among time points (*p* < 0.05), and different lowercase letters in the same row indicate significant differences among genes (*p* < 0.05).

## Data Availability

The datasets generated for this study are available on request to the corresponding author.
